# The Transdiagnostic Oncology Program (TOP): a multidomain lifestyle intervention to improve the quality of life of cancer survivors - a before-and-after pilot study in primary care

**DOI:** 10.1186/s12885-025-15063-2

**Published:** 2025-11-10

**Authors:** Sanne H. Booij, Amy Pieper, Christianne D. Wester, Ute Bültmann, Elkana C. Waarsenburg, H. J. Rogier Hoenders

**Affiliations:** 1 Center for Integrative Psychiatry, Lentis Groningen, Hereweg 76, Groningen, 9725 AG The Netherlands; 2 Rob Giel Research Center, University of Groningen, University Medical Center Groningen, Hanzeplein 1, Groningen, 9713 GZ The Netherlands; 3 Department of Health Sciences, Community and Occupational Medicine, University of Groningen, University Medical Center Groningen, Hanzeplein 1, Groningen, 9713 GZ The Netherlands; 4 Primary Care Facility Kloosterveen, Transportweg 8, Assen, 9405 PR The Netherlands; 5 Gezondheidscentrum Assen-Oost, Brunelstraat 2a, Assen, 9404 KD The Netherlands; 6 Department Comparative Study of Religion, Faculty Religion Culture and Society, University of Groningen, Oude Boteringestraat 38, Groningen, 9712 GK The Netherlands

**Keywords:** Primary-care, Aftercare, Cancer survivor, Feasibility, Transdiagnostic

## Abstract

**Background:**

There is a need for interdisciplinary primary care-led aftercare programs for the common (transdiagnostic) problems cancer survivors experience to increase their quality of life. The aim of this before-and-after (pre-post) pilot study was to examine the feasibility and preliminary effectiveness of a transdiagnostic, family doctor-led interdisciplinary program to increase quality of life in a heterogeneous group of cancer survivors.

**Methods:**

Cancer survivors (*n* = 19) followed a 12-month interdisciplinary aftercare program in the primary care setting, consisting of: family doctor consultation, exercise, mind-body therapy, sleep hygiene, nutrition recommendations, and optional psychological therapy. Eligible cancer survivors who declined participation, were recruited as controls (*n* = 16). Feasibility and acceptance were assessed through attendance and attrition rates, and an evaluation form. The primary outcome was the change in quality of life from baseline (T0) to post-intervention (T2; 12 months from baseline), as measured with the EORTC QOL-C30, covering global health status, various functional domains, and symptoms. Secondary outcomes included assessments of changes in fatigue (Multidimensional Fatigue Inventory), psychological symptoms (Depression, Anxiety and Stress scale), happiness (Happiness Index), and work ability (Work Ability Index). Intention-to-treat multilevel analyses were conducted.

**Results:**

Dropout (*n* = 3) during the program was related to personal and health issues, and attendance rates and satisfaction scores were satisfactory. At baseline, the intervention group scored significantly worse on several quality-of-life indices, and on secondary outcomes, compared to controls. The intervention group showed significantly larger increases at T2 in physical and social functioning, and decreases in fatigue and anxiety, compared to the control group.

**Conclusions:**

The results suggest that this primary care-led aftercare program is feasible and acceptable. Due to the small sample size and non-randomized design, improvements in quality of life and related outcomes should be interpreted with caution. A randomized controlled trial is warranted.

**Trial registration:**

This study was retrospectively registered at clinicaltrials.gov (NCT06809452).

**Supplementary Information:**

The online version contains supplementary material available at 10.1186/s12885-025-15063-2.

## Background

Cancer is the second leading cause of death globally [1], and the prevalence of some cancer types is rising worldwide [2]. This rise appears to be linked to unhealthy lifestyle factors, such as poor diet, lack of physical activity, overweight, sedentary behaviour, alcohol use and smoking [3, 4]. Early detection and improved treatment methods (chemotherapy, hormonal, radiation, operation) have resulted in an increased life expectancy [5]. However, surviving cancer is often associated with a reduced quality of life and impairments in both psychological and physical functioning. Transdiagnostic clinical problems of cancer survivors that lead to a reduced quality of life include pain, fatigue, anxiety and depression, fear of cancer recurrence, cognitive impairments, sleep problems and problems with returning to work [6]. In the Netherlands, almost 75% of cancer survivors (with recency of onset varying between several months to several years) reported fatigue, 30–50% reported symptoms of depression and/or anxiety, and about 50% suffer from impairments in cognitive functioning [7]. Moreover, these problems reduce the patient’s ability to return to a productive work life [8, 9], while working is viewed as a sign of well-being and normality, providing structure, social contacts, and financial support [10]. Hence, there is an urgent need for aftercare programs for cancer survivors that improve overall quality of life.

So far, these problems have been addressed in a range of aftercare programs focused on a single or a few intervention components [6]. For example, exercise programs reduce cancer-related fatigue during and after treatment [11, 12] and improve workability [13]. Furthermore, dietary and/or physical activity interventions improve physical functioning and fatigue in gynaecological cancer survivors [14], but these interventions seemed ineffective in reducing overall quality of life. An interdisciplinary approach to aftercare with a focus on healthy lifestyle, including several therapeutic disciplines and intervention components seems necessary to increase overall quality of life and address the complex and personal needs of cancer patients [15].

There are several hypothesized advantages to an interdisciplinary approach. First, cognitive, affective and sleep problems require different management strategies than the often-targeted pain and fatigue. This includes mind-body interventions and psycho-oncology [6]. Second, addressing multiple problems with different strategies may work synergistically. For example, fear of cancer recurrence might be more effectively targeted if psychological therapy is used to normalize it [16], and self-management is increased by addressing lifestyle changes that can reduce the patient’s actual risk of recurrence [17, 18]. In addition, according to the currently dominating network theory of mental disorders [19, 20], complex causal interactions between many of the reported clinical problems (e.g., anxiety, depression, fatigue, cognitive problems) are expected, and therefore, reductions in these problems are expected to reinforce reductions in others through positive upward spirals.

According to a recent review [15], inter- and multidisciplinary rehabilitation programs for cancer survivors have the potential to improve overall quality of life including return to work. However, these programs were conducted in addition to specialist-led follow-up or active treatment. Primary care-led programs (e.g., coordinated by the family doctor (FD)) have the potential to combine various kinds of follow-up care. Importantly, they have been shown to be equally effective, are widely applicable to many cancer survivors, and are more cost-effective than specialist-led care [21]. In line with this, the Dutch General Practitioner’s Association [22] recommends primary care-led interdisciplinary aftercare. Therefore, the current study investigates the feasibility and preliminary effectiveness of the “Transdiagnostic Oncology Program” (TOP), a transdiagnostic, integrative, FD-led interdisciplinary aftercare program for cancer survivors.

**Fig. 1:**
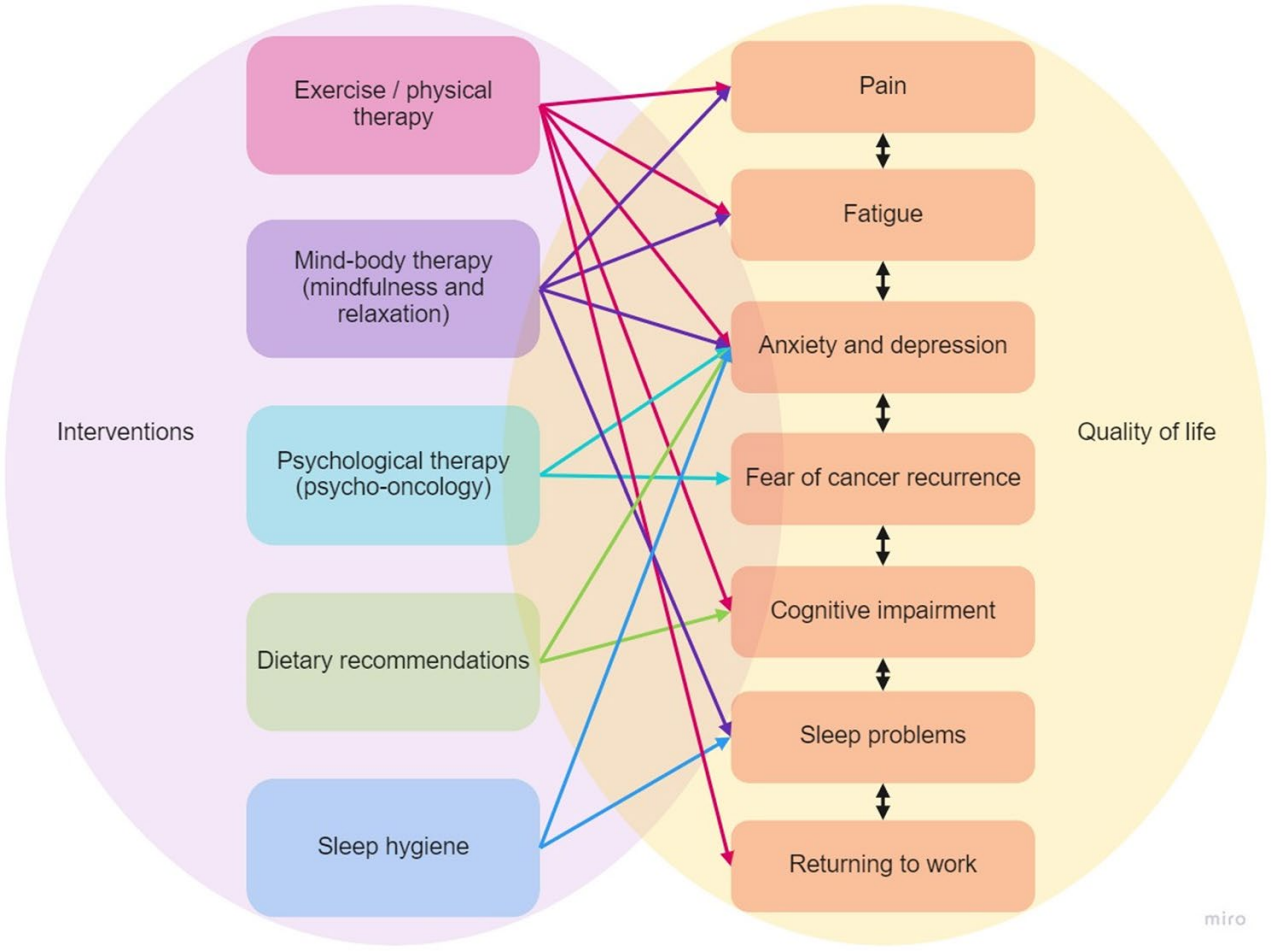
Transdiagnostic clinical problems and their (hypothesized) complex interrelations with intervention components. Note. Sources: Emery et al., 2022 and Wilson et al., 2022. We acknowledge that recent evidence suggests additional associations between components, for example, there is an association between physical activity and sleep problems [24]. However, as TOP was designed with the recommendations of Emery et al., (2022) in mind, we decided not to include these arrows in the figure

TOP is a 12-month multidomain lifestyle intervention aimed at improving overall quality of life, by targeting the most common (transdiagnostic) clinical problems of cancer survivors (see Fig. 1). In a smaller, single-centre, uncontrolled pilot study, Beumeler et al. [23] showed that TOP was feasible and that quality of life, among others, improved within the intervention group. The current study re-examines the feasibility and preliminary effectiveness of TOP in a larger, multicentre, controlled before-and-after (pre-post) pilot study. Results of this study will inform further refinements to the program and help to determine the suitability of a future full-blown randomized controlled trial. It was hypothesized that, in line with the previous pilot study, TOP is feasible in the primary care setting. Furthermore, it was hypothesized that, due to its multiple components and interdisciplinary nature, TOP leads to improvements in quality of life in the intervention, compared to the control group. Secondary outcomes included the assessment of intermediate outcomes important for quality of life (happiness, fatigue, mental health, work ability). These were also hypothesized to improve in the intervention group compared to the control group, as they have previously been shown to be targeted by one or more of the intervention components [6].

## Methods

### Design & participants

**Fig. 2:**
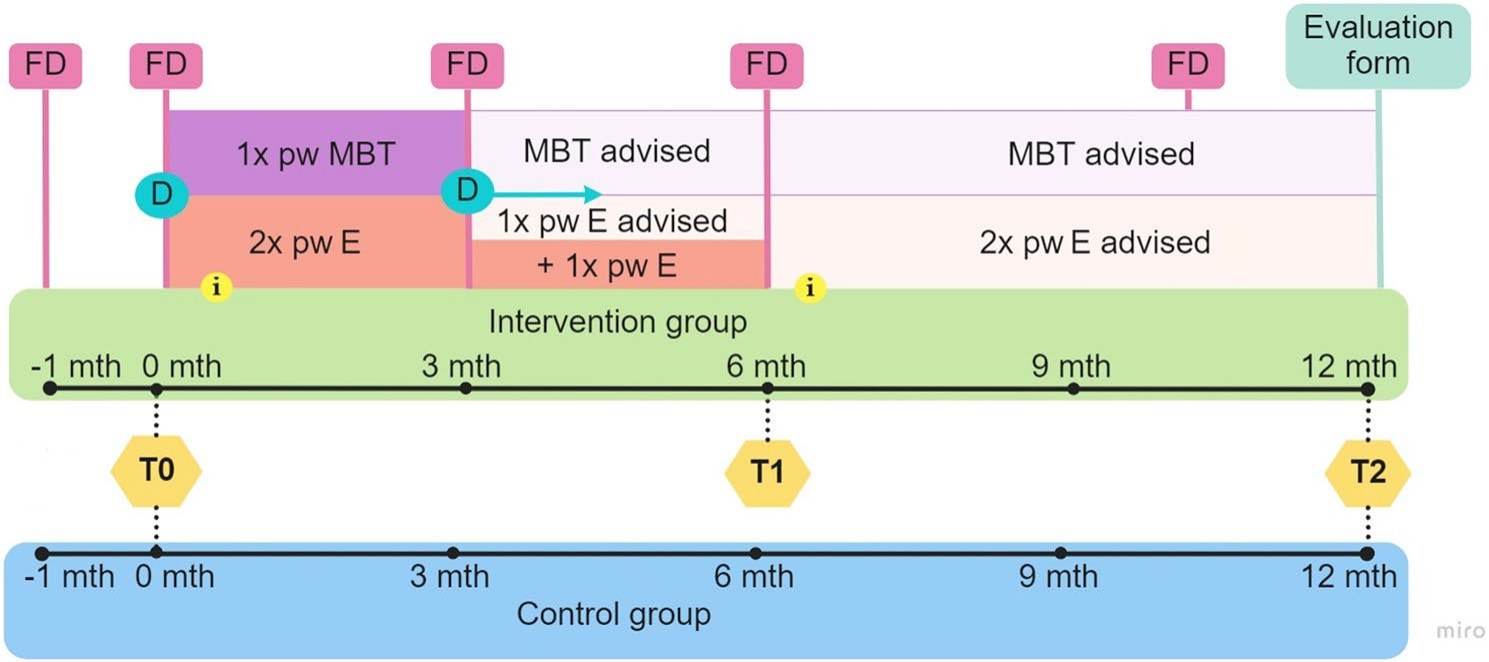
An overview of the intervention and study design. Note. Time scheme of the program for the intervention group and the control group. Including exercise (E), mind-body therapy (MBT), two group informational sessions about nutrition and sleep hygiene (i), the four individual meetings with the family doctor (FD) to evaluate the process (the meeting before intervention was for screening), the individual meetings with the dietician (D) and the assessment waves (T). Meetings with the dietician were, from the second meeting on, optional, depending on personal need

This study was an observational study with a pretest-posttest design within the context of routine care, in which data were collected on the effectiveness of an existing program. The control group consisted of patients who chose not to participate in the program. This design was chosen over a randomized controlled trial due to practical constraints (e.g., limited financial and logistical resources) and the goal to study the intervention under real-world conditions to enhance the ecological validity. Although randomization was not possible, a comparison group was included to strengthen causal inference relative to a single-group pre-post design. Figure 2 shows the study design, with three assessments^1^: pre-intervention, at baseline (T0), mid-intervention, 6 months after baseline (T1), and post-intervention, 12 months after baseline (T2). Outcome measures were assessed at all three assessment waves. While T0 and T2 were used to assess preliminary effectiveness, the mid-intervention assessment was used to examine the timing of changes within the intervention group. In addition, feasibility and satisfaction with the intervention were assessed at T2, using an evaluation form.

Participants were recruited for TOP via an e-referral program (‘Zorgdomein’), local newspapers and flyers in the waiting room of eleven primary care clinics. In addition, two of them (Kloosterveen and Gezondheidscentrum Assen-Oost, Assen, the Netherlands) screened their caseload for potentially eligible participants by using the International Classification of Primary Care (ICPC) code for oncology. Inclusion criteria for TOP were (1) being between ≥ 18 and ≤ 75 years old, and (2) having been diagnosed with cancer at least 6 months ago and/or having been long-term stable. Exclusion criteria included (1) the presence of severe physical risks due to cancer treatment and/or comorbidities, (2) currently undergoing intensive chemotherapy or other treatment, (3) having neuropsychiatric disorders that would severely hinder participation, such as psychosis or dementia (4) having skin cancer, except for melanomas with metastasis, and (5) having a life expectancy of < 1 year. In- and exclusion criteria for the study were the same, with the addition that the participant was willing to provide written informed consent.

**Fig. 3:**
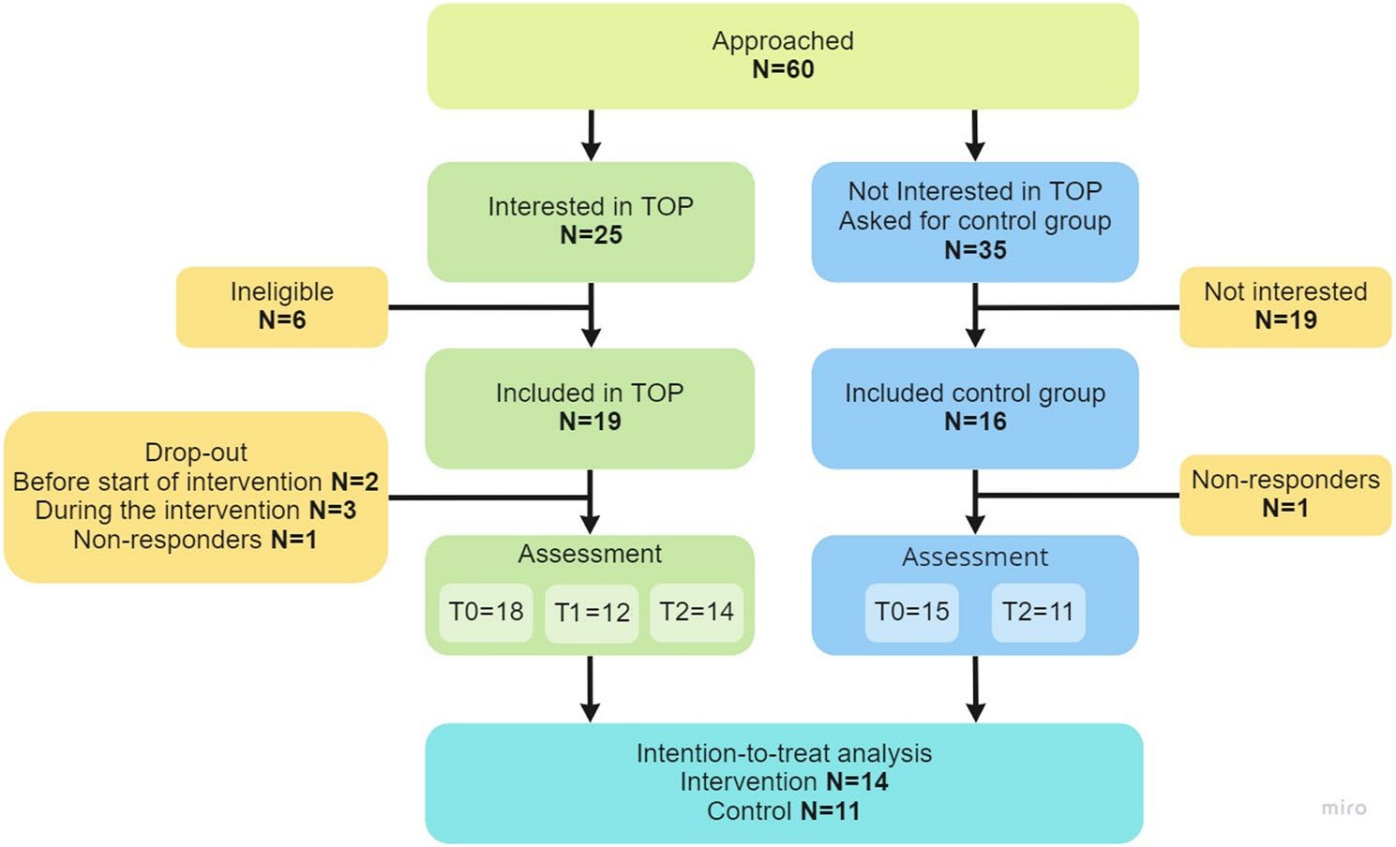
Recruitment and participation flowchart. Note. Two participants in the intervention group dropped out before the start, whereof one kept filling out questionnaires. This person was included in the analysis (intention-to-treat)

Sixty potential participants were approached by the FD. The participants who were eligible and interested were called the ‘intervention group’ (*n* = 19) and allocated to one of the two training locations based on where they lived. The others (*n* = 35), who indicated no interest, were asked to serve as part of a convenience control group, called the ‘control group’. Sixteen of them agreed to do so (shown in Fig. 3). All participants agreed to participate and signed an informed consent form before starting the study. The Medical Ethical Committee of the University Medical Centre Groningen judged the protocol (M17.218911) to be exempted from review by the Medical Research Involving Human Subjects Act (in Dutch: WMO).

### Intervention (exposure)

The “Transdiagnostic Oncology Program” (TOP) is a 12-month interdisciplinary aftercare program designed to enhance the quality of life of cancer survivors, by addressing common (transdiagnostic) clinical problems. The current study monitored the program that ran between January 2019 and January 2020 [23]. During the first three months, the emphasis was on exercise to increase strength and fitness, guided by the physiotherapists. The participants met twice a week for a 1-hour exercise (E) session. Once a week, the exercise session was followed by a 1-hour mind-body therapy (MBT) to reduce stress by learning relaxation and mindfulness techniques. In addition, a group informational meeting about nutrition and cancer (i) was provided by the dietician (D) around the start of the program. Afterwards, participants were advised to make at least two appointments with a dietician about making healthy food choices with practical advice on cooking and eating habits. Depending on personal need, participants could make more appointments with the dietician.

The supervised exercise training consisted of both cardio (± 25 min) and muscle strength training (± 35 min). The specific training schedule was personalized using results from the Steep Ramp test and Resistance Machine tests (e.g., leg press, vertical traction, leg extension). At week 8 and week 14, the schedule was adapted, again based on these tests. Cardio training consisted of cycling (intervals), and depending on the results of the tests, also a treadmill or ergometer. Strength training consisted of 20 exercises using fitness equipment, such as leg extension, leg curl, and vertical traction, except when these were not possible due to physical complaints. In addition, exercises were performed aimed at improving stability and coordination, in both closed and open chains. For strength training, small adaptations were also made on a session-by-session basis.

Between 3 and 6 months, exercise was reduced to once a week 1 h. Besides this 1 h session, participants were urged to exercise at least 1 h once a week and continue to do relaxation exercises. This gradual reduction aims to incorporate sports and relaxation activities into the patient’s daily life in a sustainable way [25, 26]. In addition, a second informational group meeting (i) was given about stress and sleep hygiene by the FD and the physiotherapist.

After the 6th month, participants were supposed to continue exercising and perform relaxation activities in their own time. Further, each participant met with the FD 4 times a year to evaluate their progress, personal goals and mild psychological problems. Lastly, for the participants who experienced significant psychological problems, sessions with a psychologist at an institute specialized for psycho-oncology, were available. All intervention components, their frequency, duration and mode are summarized in Supplementary Table S1.

### Assessments

Assessments included self-report questionnaires administered by the FD. For the intervention group, measurements were done at all three assessment waves, unless noted (Supplementary Table S2 shows a measurement overview). The control group participated in the FD-administered (self-report) assessments at baseline (T0) and post-intervention (T2). At every measurement, the FD sent an email including a secured link (Roqua; www.roqua.nl) to all the questionnaires, which took about 40–45 min to fill in. If necessary, a reminder was sent. The assessments are described briefly below.

Feasibility and satisfaction. Feasibility of the intervention was assessed through attrition and attendance rates and responses to an evaluation form at post-intervention (T2), including adverse effects, which can be found in Supplementary Text S1. Serious adverse events were also monitored by the FD. Satisfaction with the intervention was assessed with the same evaluation form at T2.

Primary outcome. Quality of life (QoL) was measured with the validated European Organization for Research and Treatment of Cancer 30-item Quality of Life Questionnaire (EORTC QLQ-C30) [27]. This included global health status, an overall indicator of quality of life, five functioning scales (physical, emotional, cognitive, social and role) and three symptom scales (fatigue, nausea and vomiting, and pain).

Secondary outcomes. Fatigue was comprehensively assessed by the validated Dutch version of the Multidimensional Fatigue inventory (MFI); the MFI-20 [28]. Psychological symptoms were assessed with the 21-item Depression, Anxiety and Stress Scale (DASS-21), which is a reliable and validated questionnaire [29, 30]. Happiness, a specific aspect of quality of life, was measured with the happiness index (HI) [31]. Work ability was assessed by a validated single-item question from the work ability index (WAI) [32]. Work accommodations after work resumption was assessed with six contextual items [33]. Participants who resumed work at any of the assessments (T0, T1, T2) were asked to specify any work accommodations.

### Data analysis

Baseline demographics and clinical characteristics were described and compared between the intervention and control group and between the two training locations, using Pearson’s chi-square tests, independent-samples T tests (with bootstrap correction) and Mann-Whitney U-tests, depending on the data type and distribution.

(Preliminary) effects on the primary and secondary outcome variables were examined with (intention-to-treat) multilevel analyses. This was chosen because of the hierarchical structure of the data. The outcome measures were used as dependent variables with the group (intervention/control) as between-subjects factor and time (measurement T0 and T2) as the within-subject factor. In this way, it was possible to examine the Group X Time interaction to assess whether the change over time differed between the groups. See Supplementary Text S2 for multilevel models specification.

As the intervention group had an additional assessment halfway the intervention (T1), multilevel analyses were also used to assess within-subject change during the study period (intervention group only). This allows for a more in-depth view on the timing of changes within the intervention group. The outcome measures were used as dependent variables with time (dummy-coded) as the within-subject change factor. For this, changes from baseline (T0) to the mid- (T1) and post-intervention (T2) were examined. To further characterize these within-group changes, the multilevel analysis version of Cohen’s d (Cohen’s dr) was calculated for each of the outcomes, with effect sizes of 0.20, 0.50, and 0.80 indicating small, medium and large effects, respectively [34, 35].

For the multilevel analyses, bootstrapping with 1000 replications was performed for the models with clearly non-normally distributed residuals (according to QQ plots) and/or high skewness or kurtosis. Light tails were accepted. Heteroskedasticity was examined by plotting residuals versus predicted values. None was detected. For all analyses, differences were considered statistically significant when *p* < 0.05. As this was a pilot study, examining preliminary effectiveness, we did not adjust our p-values for multiple testing in our analyses. The score for nausea of the EORTC QLQ C-30 was excluded from the statistical analyses because this symptom was not experienced by many of the cancer survivors, which led to highly skewed and 0-inflated data. Work accommodations were only assessed within individuals who resumed work and was therefore only assessed descriptively. Because we utilized a non-randomized convenience control group, there might be systematic differences between the intervention and control groups, leading to bias in the results. Therefore, in case of baseline differences between the groups, a posthoc analysis with correction for starting levels would be conducted to minimize this bias (although bias cannot fully be mitigated in this design).

### Power

As this was a pilot study, the sample size was not determined based on power, but rather on the size needed to establish feasibility, and the maximum number of participants that could be included in the intervention group, given constraints, such as location and staff size.

## Results

### Feasibility of and satisfaction with the program

In the intervention group, two individuals dropped out before the start of the intervention, and three dropped out due to personal (bereavement) or health issues (psychological problems; recurrent cancer), see Fig. 3. The attendance rates were: PE 75%; MBT 77%; FD and dietician 100%; food & cancer 88%; stress & sleep 63%. Meetings with the dietician were optional after the second time and participants attended on average 4 to 5 sessions (range 2–9). The optional sessions for psycho-oncology were requested by six (32%) participants of which three participants already went before the program. No serious adverse events related to TOP were reported. However, four participants were temporarily (partly) absent during the intervention period, due to illness/infection (*n* = 2), surgery (*n* = 1) and chemotherapy (*n* = 1). One person reported adverse effects, specifically muscle soreness and emotional effects, described as a necessary but emotionally difficult confrontation with their condition. 85% of the participants thought the program was feasible and found the duration of the program just right. Most of the participants reported they had sufficient practical tools with respect to exercise (92%), nutrition (85%) and relaxation (62%) after the intervention. In terms of sustainability of the program, the majority (69%) said they had been able to keep up the lifestyle changes they had built up in the first six months. Finally, the burden of the assessments, on a scale from 1 to 10, was rated on average 3.85 (SD = 2.64).

Regarding overall satisfaction with the program, participants rated the program 8 out of 10 (SD = 0.80). Additionally, participants rated the improvement in quality of life 8 out of 10 (SD = 1.12) and the added value of the lifestyle intervention being multidimensional 9 out of 10 (SD = 1.09). The participants were also satisfied with the program content (course material and information meetings); 4 out of 5. Most participants found the frequency of the meetings with the physiotherapy (61%), FD (92%) and dietician (85%) just right, while the majority found the frequency of the relaxation (mind-body) meetings too low (55%). All but one participant (92.3%) would recommend the program to others; one participant said ‘maybe’. All other feasibility outcomes from the evaluation questionnaire can be found in Supplementary Table S3.

### Sample characteristics & group similarity

No significant differences in age and gender were observed between intervention and control (see Table 1). Participants were treated for various types of cancer; breast and rectal/colon cancer were most common in both groups. The intervention group had significantly worse scores on various outcome variables at baseline, compared to control. For the intervention group, the results of the participants from the two training locations did not differ.

**Table 1:** Baseline characteristics of the intervention and control group

	Baseline				
	Control group		Intervention group		
	*N*	(%)	*N*	(%)	*P*-value
Gender					0.713
Female	10	(62.5)	13	(68.4)	
Male	6		6		
Cancer type					^-A^
Bladder cancer	1	(6.3)	-	-	
Breast cancer	6	(37.5)	9	(47.4)	
(non) Hodgkin lymphoma	2	(12.5)	-	-	
Kidney cancer	-	-	1	(5.3)	
Laryngeal cancer	-	-	1	(5.3)	
Leukemia	-	-	1	(5.3)	
Prostate cancer	1	(6.3)	2	(10.5)	
Rectal/colon cancer	5	(31.3)	4	(21.2)	
Testicular cancer	-	-	1	(5.3)	
Vaginal cancer	1	(6.3)	-	-	
Work status^a^					-^A^
Retirement	5	(33.3)	6	(23.1)	
Paid work	5	(33.3)	6	(23.1)	
Unpaid work	3	(20.0)	10	(38.5)	
Benefits	2	(13.3)	4	(15.4)	
	Mean	SD	Mean	SD	*P*-value
Age (years)	60.0	12.5	59.5	11.4	0.420
Quality of Life (EORTC QLQ-C30) (0–100)					
*Functioning* ^*a*^					
Physical functioning	91.67	20.87	80.37	14.55	0.001*
Role functioning	87.50	15.51	68.52	19.71	0.005*
Emotional functioning	87.50	16.39	66.67	22.32	0.003*
Cognitive functioning	86.46	13.90	73.15	19.08	0.032*
Social functioning	93.75	8.34	71.30	25.44	0.008*
*Symptoms* ^*b*^					
Fatigue	22.22	16.73	44.45	22.87	0.003*
Nausea and vomiting	2.08	8.33	2.78	11.79	0.966
Pain	7.29	18.23	22.22	25.56	0.055
Global health status^c^	84.90	11.87	61.57	16.20	0.000*
*Fatigue (Multidimensional Fatigue Inventory) (4–20)* ^*d*^					
General fatigue	9.73	4.35	15.1	4.51	0.003*
Physical fatigue	8.20	3.73	13.8	5.26	0.003*
Reduced activity	9.67	4.66	12.6	4.29	0.091
Reduced motivation	7.33	3.92	10.8	4.23	0.023*
Mental fatigue	7.33	3.35	13.1	4.59	0.001*
*Mental symptoms (Depression, Anxiety and Stress Scale-21) (0–42)* ^*e*^					
Depression	2.13	2.67	6.56	6.64	0.024*
Anxiety	1.73	1.98	6.22	6.13	0.024*
Stress	2.67	2.89	11.11	8.49	0.002*
Happiness (Happiness index) (1–10)^h^	8.36	1.28	5.17	2.53	0.000*
Work ability (Work ability index) (0–10)^i^	5.38	3.12	4.12	2.47	0.152
Work related functioning (5-item WRFQ v2.0) (0–100)^j^	79.75	12.39	60.00	28.28	-^A^

The sample size for the control group varies between *N* = 13 and *N* = 16 and for the intervention group between *N* = 15 and *N* = 18, except for work functioning (*N* = 5 for the control group and *N* = 2 for the intervention group). WRFQ = Work related functioning questionnaire

^a^ higher score indicates better health-related quality of life

^b^ higher score indicates worse health-related quality of life

^c^ higher score indicates better health-related quality of life

^d^ higher score indicates more subjective fatigue

^e^ higher score indicates greater levels of distress on each subscale

^f^ higher score indicates more grip strength

^g^ higher score indicates more pain

^h^ higher score indicates more happiness

^i^ higher score indicates better work ability

^j^ higher scores indicates better work functioning

*Significant change between groups (*p* < 0.05)

^A^ Due to the small sample size no statistical test is possible

### Between group differences from baseline to post-intervention

Regarding the primary outcomes, significant differences were found between the two groups in the change from baseline to post-intervention for physical functioning, social functioning, and fatigue on the quality-of-life questionnaire (Table 2). Specifically, the intervention group increased in physical and social functioning and decreased in fatigue, whereas the control group stayed relatively stable. Significant differences were also found for secondary outcomes; the intervention group showed significant reductions in general, physical and mental fatigue, and anxiety, compared to the control group, which again stayed relatively stable.

**Table 2:** Intention-to-treat multilevel analyses to compare the change in outcome variables between the intervention and control group

		B	95% C.I		P -value
			Lower	Upper	
**Primary outcomes**					
*Quality of life (EORTC QLQ-C30)*					
Global health status	Baseline	84.90	77.41	92.38	0.000*
	Time	0.22	−8.08	8.52	0.957
	Group	−23.32	−33.61	−13.04	0.000*
	Interaction	10.49	−0.53	21.50	0.061
*Functioning scale*					
Physical functioning	Baseline	91.66	83.15	100.18	0.000*
	Time	−2.40	−8.08	3.29	0.395
	Group	−11.30	−23.00	0.41	0.058
	Interaction	9.86	2.35	17.37	0.012*
Role functioning ^a^	Baseline	87.50	76.13	96.50	0.001*
	Time	−3.90	−25.85	17.76	0.794
	Group	−18.98	−31.40	−4.83	0.004*
	Interaction	17.24	−10.27	39.69	0.309
Emotional functioning	Baseline	87.50	77.64	97.36	0.000*
	Time	−1.17	−10.70	8.37	0.803
	Group	−20.83	−24.39	−7.28	0.003*
	Interaction	5.36	−7.28	18.00	0.390
Cognitive functioning	Baseline	86.46	78.05	94.86	0.000*
	Time	1.70	−7.48	10.88	0.707
	Group	−13.31	−24.86	−1.76	0.025
	Interaction	1.71	−10.47	13.89	0.776
Social functioning	Baseline	93.75	85.47	102.03	0.000*
	Time	0.64	−10.40	11.67	0.907
	Group	−22.45	−33.83	−11.08	0.000*
	Interaction	21.09	6.37	35.80	0.006*
*Symptom scale*					
Fatigue	Baseline	22.22	12.79	31.65	0.000*
	Time	1.93	−6.85	10.71	0.656
	Group	22.23	9.27	35.18	0.001*
	Interaction	−19.65	−31.27	−8.02	0.002*
Pain	Baseline	7.29	−3.40	17.98	0.176
	Time	7.67	−2.75	18.09	0.143
	Group	14.93	0.24	29.62	0.047*
	Interaction	−13.22	−27.03	0.58	0.060
**Secondary outcomes**					
*Fatigue (Multidimensional Fatigue Inventory)*					
General Fatigue	Baseline	9.73	7.41	12.05	0.000*
	Time	0.73	−1.49	2.95	0.508
	Group	5.32	2.18	8.46	0.001*
	Interaction	−3.95	−6.89	−1.02	0.010*
Physical Fatigue	Baseline	8.20	5.83	10.57	0.000*
	Time	1.17	−0.74	3.08	0.219
	Group	5.63	2.43	8.84	0.001*
	Interaction	−3.74	−6.26	−1.22	0.005*
Reduced Activity	Baseline	9.67	7.37	11.96	0.000*
	Time	−0.53	−2.33	1.27	0.550
	Group	2.89	−0.21	5.99	0.067
	Interaction	−1.56	−3.93	0.82	0.190
Reduced Motivation	Baseline	7.33	5.31	9.36	0.000*
	Time	0.15	−1.53	1.82	0.860
	Group	3.50	0.76	6.24	0.014*
	Interaction	−2.04	−4.25	0.17	0.069
Mental Fatigue	Baseline	7.33	5.33	9.33	0.000*
	Time	0.94	−0.78	2.66	0.271
	Group	5.78	3.07	8.48	0.000*
	Interaction	−2.52	−4.79	−0.25	0.031*
*Mental symptoms (Depression*,* Anxiety and Stress Scale)*					
Depression ^a^	Baseline	2.13	1.07	3.46	0.000*
	Time	−0.08	−2.18	2.03	0.942
	Group	4.42	1.42	6.29	0.004*
	Interaction	−1.07	−4.04	2.57	0.627
Anxiety	Baseline	1.73	−0.53	4.00	0.129
	Time	0.17	−0.11	1.39	0.775
	Group	4.49	1.42	7.55	0.005*
	Interaction	−2.29	−3.90	−0.68	0.007*
Stress	Baseline	2.67	−0.75	6.09	0.123
	Time	1.06	−1.92	4.02	0.470
	Group	8.44	3.82	13.07	0.001*
	Interaction	−3.60	−7.50	0.33	0.071
*Happiness (Happiness Index)*					
Happiness ^a^	Baseline	8.36	7.80	8.89	0.001*
	Time	−0.12	−1.00	0.80	0.837
	Group	−3.19	−3.94	−1.99	0.001*
	Interaction	0.67	−0.84	1.79	0.504
*Work-related functioning (Work Ability Index)*					
Work ability	Baseline	5.39	3.90	6.87	0.000*
	Time	0.53	−1.11	2.17	0.511
	Group	−1.27	−3.24	1.09	0.202
	Interaction	1.01	−1.27	3.30	0.370

*Significant *p*-value

^a^ If the residuals were skewed, a bootstrapped analysis was performed. The confidence interval and *p*-value are from the bootstrapped analysis.

A posthoc analysis with correction for starting levels was conducted to account for potential selection bias. The results are presented in Supplementary Table S4. In short, all but two outcomes, namely social functioning and mental fatigue, were still (trend) significant.

### Within-intervention group changes from baseline to mid and post-intervention

To provide a more in-depth understanding of the nature and timing of changes within the intervention group, the within-group changes, and the accompanied effect size, from baseline to mid- and post-intervention were assessed (Table 3). Regarding the primary outcomes that showed significant differences for the between-group comparisons; physical and social functioning increased non-significantly from baseline to mid-intervention, with small to moderate effects, respectively. At post-intervention, the changes reached significance and were moderate and large, respectively. A distinct pattern was observed for fatigue; fatigue showed a significant (moderate) decrease from baseline to mid-intervention, which became a large decrease at post-intervention. The same pattern emerged for the secondary outcome measures general and physical fatigue, but not for mental fatigue, which generally showed smaller effects, and only reached significance at post-intervention. Finally, anxiety showed a non-significant decrease (moderate effect) from baseline to mid-intervention. At post-intervention, this effect became large and significant.

**Table 3:** Intention-to-treat multilevel analyses of the within-individual change trajectories for the intervention group

		B	95% C.I.		P-value	Effect Size d_r_^b^
			*Lower*	*Upper*		
**Primary outcomes**						
*Quality of life (EORTC QLQ-C30)*						
Global health status	T0	61.57	53.59	69.56	0.000*	-
	T1	8.70	1.33	16.08	0.023*	0.53
	T2	10.97	2.24	19.70	0.015*	0.66
*Functional scales*						
Physical functioning	T0	80.37	73.67	87.07	0.000*	-
	T1	4.30	−3.32	11.92	0.257	0.26
	T2	8.78	−0.24	17.31	0.044*	0.53
Role functioning	T0	68.52	58.79	78.25	0.000*	-
	T1	4.69	−8.55	17.92	0.475	0.23
	T2	13.56	−0.21	27.32	0.053	0.66
Emotional functioning	T0	66.67	55.89	77.45	0.000*	-
	T1	7.67	−0.66	16.00	0.069	0.35
	T2	4.75	−5.43	14.94	0.349	0.21
Cognitive functioning	T0	73.15	63.28	83.01	0.000*	-
	T1	−4.69	−17.30	7.92	0.453	0.23
	T2	3.49	−10.01	16.99	0.605	0.17
Social functioning	T0	71.30	61.62	80.97	0.000*	-
	T1	8.66	−1.87	19.18	0.103	0.43
	T2	21.74	9.79	33.69	0.001*	1.08
*Symptom scales*						
Fatigue	T0	44.45	34.77	54.12	0.000*	-
	T1	−11.30	−18.20	−4.36	0.002*	0.57
	T2	−17.81	−26.40	−9.22	0.000*	0.89
Pain	T0	22.22	11.64	32.81	0.000*	-
	T1	−2.04	−11.57	7.49	0.663	0.09
	T2	−5.56	−16.90	5.79	0.327	0.25
**Secondary outcomes**						
*Fatigue (Multidimensional Fatigue Inventory)*						
General Fatigue	T0	15.06	12.81	17.30	0.000*	-
	T1	−2.53	−4.00	−1.06	0.001*	0.55
	T2	−3.19	−5.04	−1.35	0.001*	0.69
Physical Fatigue	T0	13.83	11.40	16.26	0.000*	-
	T1	−1.96	−3.34	−0.58	0.007*	0.39
	T2	−2.56	−4.32	−0.80	0.006*	0.51
Reduced Activity	T0	12.56	10.58	14.53	0.000*	-
	T1	−2.06	−3.44	−0.68	0.005*	0.51
	T2	−2.10	−3.81	−0.38	0.018*	0.52
Reduced Motivation	T0	10.83	8.97	12.69	0.000*	-
	T1	−2.13	−3.74	−0.52	0.011*	0.55
	T2	−2.10	−4.02	−0.17	0.034*	0.55
Mental Fatigue	T0	13.11	11.00	15.22	0.000*	-
	T1	−0.90	−2.32	0.54	0.211	0.21
	T2	−1.60	−3.39	0.18	0.077	0.37
*Mental symptoms (Depression*,* Anxiety and Stress Scale)*						
DASS – Anxiety	T0	6.22	3.50	8.94	0.000*	-
	T1	−1.22	−2.74	0.29	0.109	0.22
	T2	−2.26	−4.19	−0.33	0.024*	0.41
DASS – Depression ^a^	T0	6.56	3.51	9.60	0.000*	-
	T1	−1.31	−4.06	2.09	0.480	0.23
	T2	−1.10	−3.73	1.73	0.473	0.19
DASS – Stress	T0	11.11	7.24	14.98	0.000*	-
	T1	−2.74	−6.03	0.55	0.099	0.35
	T2	−3.00	−6.95	0.94	0.131	0.38
*Happiness (Happiness Index)*						
Happiness	T0	5.17	3.95	6.38	0.000*	-
	T1	0.80	0.09	1.50	0.028*	0.32
	T2	0.50	−0.41	1.41	0.273	0.20
*Work-related functioning (Work Ability Index)*						
Work ability	T0	4.12	2.79	5.45	0.000*	-
	T1	1.19	−0.11	2.49	0.070	0.44
	T2	1.62	−0.03	3.28	0.054	0.60

^a^ The residuals were skewed. A bootstrapped analysis was performed. The confidence interval and p-value are from the bootstrapped analysis

^b^ Effect size (dr) was calculated by dividing the estimates at T1/T2 (ignoring their sign) by the square root of the residual variance at T1 or T2 giving a mixed model regression version of Cohen’s d

*Significant p-value as compared to baseline.

### Work-related functioning

All but one individual in the intervention group increased their working hours from baseline to post-intervention, while all individuals in the control group stayed stable (see Supplementary Figure S1).

## Discussion

The current study investigated the feasibility and preliminary effectiveness of a transdiagnostic, integrative, FD-led interdisciplinary aftercare program for cancer survivors, called the “Transdiagnostic Oncology Program” (TOP). Results indicate that the TOP program was feasible and acceptable in a (multicenter) primary care setting in the Netherlands, for a heterogeneous group of cancer survivors. In addition, the improvements in physical and social functioning, fatigue, and reduction in anxiety in the intervention group, as compared to the control group, cautiously suggest that this interdisciplinary program improves the overall quality of life of cancer survivors.

The feasibility and acceptance were supported by high attendance and completion rates, and by all participants being satisfied with the overall program. Like the previous smaller, non-controlled, pilot [23], participants felt the frequency of the mind-body therapy was too low. This might have contributed to the finding that relatively few participants (65% compared to 92% for exercise and 85% for nutrition) indicated that they had sufficient practical tools with respect to relaxation. Offering mind-body therapy for a longer period and/or more frequently, might also lead to a stronger improvement in emotional functioning and other mental health outcomes.

Self-reported maintenance of lifestyle changes after one year seems relatively high (69%), given that there was a large decrease in supervision and activities during the second half of the program; participants were supposed to continue exercising and performing relaxation activities on their own, while maintaining low frequent contact with the FD. According to a systematic review of Spark et al. [36]. into maintenance of physical activity and/or dietary interventions, from the few studies that assessed post-intervention maintenance of outcomes, only 40% achieved successful maintenance 3-months post-intervention. Given that most of the quality of life-related outcomes continued to improve during the second half of the year, this supports the notion that the program was relatively successful in achieving sustainable lifestyle changes.

The finding that the intervention group, compared to the control group, showed a reduction in several aspects of quality of life, including physical, and social functioning, and fatigue, as measured with the EORTC QLQ-C30, as well as secondary outcomes such as anxiety, is in line with calls for more interdisciplinary programs [6, 21]. Another Dutch program that focused on two intervention components (exercise and personal counselling for work related issues), also showed improvements on several aspects of quality of life [37]. Two other studies based in Italy examined multidisciplinary approaches in breast cancer survivors, and similarly found positive effects on overall quality of life, as well as some of its subcomponents [38, 39]. However, these programs were carried out without a control group, and some started during treatment, making it difficult to tease apart effects from the intervention and natural course [40]. Although our study included a control group, baseline differences and the small sample size warrant cautious interpretation of the findings. As a result, there is still limited overall evidence supporting the effectiveness of multidisciplinary aftercare programs.

It must be noted that not all aspects of quality of life improved (intervention versus control), especially the cognitive and emotional functioning scales, as well as related secondary outcomes, such as depression and happiness showed small effects, with sometimes better outcomes mid- compared to post-intervention. When looking at the baseline values of the intervention group, participants reported clinically relevant problems for emotional and cognitive functioning, according to the thresholds for clinical importance (TCIs) [41]. Hence, the intervention group had clear need for improvement on these aspects. At post-intervention, the subscale scores improved to the extent that the participants just met the threshold for no longer having a clinically important problem in these domains, but individual differences were large. More frequent mind-body sessions and more tools for implementation might help to better target these outcomes. Additionally, only about one-third of the participants chose to visit the psycho-oncologist, despite the scientific evidence for the effectiveness of psychotherapy for emotional functioning problems [6]. This might be stimulated more, by explicitly advising at least one introductory session. Collectively, the feasibility results indicate areas for further improvement.

The implementation of interventions on return-to-work (RTW) processes and work ability in cancer survivors has become an important target in cancer rehabilitation. De Boer et al. [42] systematically reviewed the effectiveness of interventions aimed at enhancing RTW in cancer patients. Five RCT’s showed moderate evidence that multidisciplinary interventions involving physical, psycho-educational and/or vocational components led to higher RTW rates than care as usual [43–47]. A recent systematic review shows that physical activity interventions consisting of 50–60 min per session of moderate to vigorous exercise twice a week, improved the RTW in cancer survivors [13]. Although return-to-work was not explicitly targeted, we hypothesized improvements in this domain due to the program’s broader effects. Our findings were mixed. According to the descriptive findings, it seems that working individuals in the intervention group showed greater improvements in working hours compared to the control group. Although work ability seemed, on average, to have improved in the intervention group, this change was not significantly different from the control group. The addition of an explicit vocational part (e.g., vocational counseling) to this interdisciplinary program, as suggested by a systematic Cochrane review [42], is currently being implemented.

A strength of this pilot study was the strong embedding of the intervention in clinical practice which ensures ecological validity. Several limitations must also be kept in mind when interpreting the findings. First, the participants were not randomized into an intervention and control group. The control group mainly consisted of participants who did not feel the need to participate. This seems to have led to selection bias; the baseline results indicate that the intervention group scored significantly worse for most of the quality of life-related outcomes compared to the control group. This means that biases such as regression towards the mean are a realistic scenario. Nevertheless, most outcomes were still (trend) significant after a posthoc correction for starting levels. Relatedly, we did not control for the stage of the disease in the analysis. In an advanced stage, the symptoms, such as fatigue, may deteriorate, even with an intervention, hence the stage may have an influence on the eventual outcome. To limit this effect, we only included individuals with a life expectancy of at least a year. Finally, we assessed lifestyle change and sustainability with self-report questionnaires, which are less valid than, for example, objective tracking devices, although these also have their limitations [48]. Future research should evaluate the improved program using a sufficiently powered randomized controlled trial, including cost effectiveness assessments and lifestyle tracking.

## Conclusions

TOP seems feasible and acceptable for a heterogeneous group of cancer survivors. Quality of life appeared to improve across multiple domains, but due to the design, this cannot be attributed to the intervention with certainty. Therefore, with small adaptations to the intervention in its current form, a larger, randomized controlled trial into the (cost-) effectiveness seems warranted to examine its value for routine (after)care in the Netherlands.

## Supplementary Information

Supplementary Material 1: Table S1. Summary of intervention components.

Supplementary Material 2: Table S2. An overview of the measurements.

Supplementary Material 3: Text S1. Evaluation form.

Supplementary Material 4: Text S2. Specification of the multilevel models.

Supplementary Material 5: Table S3. All results of the evaluation questionnaire.

Supplementary Material 6: Table S4. Posthoc Intention-to-treat multilevel analyses to compare the change in outcome variables between the intervention and control group.

Supplementary Material 7: Figure S1. Work-related functioning.

## Data Availability

The datasets generated and/or analysed during the current study are not publicly available due to the protection of participants’ privacy but are available from the corresponding author on reasonable request.

## Footnotes

1 The original study also includes an additional screening assessment one month before baseline (T-1). As this assessment is not used in the current paper, for clarity reasons, it is omitted in the description of the design.

## References

[1] Wiseman MJ. Nutrition and cancer: prevention and survival. Br J Nutr. 2018;122(5):481–7. 10.1017/S0007114518002222. 30213279 10.1017/S0007114518002222

[2] Sung H, Ferlay J, Siegel RL, et al. Global cancer statistics 2020: Globocan estimates of incidence and mortality worldwide for 36 cancers in 185 countries. CA Cancer J Clin. 2021;71(3):209–49. 10.3322/caac.21660. 33538338 10.3322/caac.21660

[3] American Institute for Cancer Research (AICR). New Study Links Cancer and cancer deaths to lifestyle factors. Available at: https://www.aicr.org/news/new-study-links-cancer-and-cancer-deaths-to-lifestyle-factors/. Accessed 15 Dec 2022.

[4] World Health Organization. Cancer. Available at: https://www.who.int/news-room/fact-sheets/detail/cancer. Accessed 15 Dec 2022.

[5] Siegel R, DeSantis C, Virgo K, et al. Cancer treatment and survivorship statistics, 2012. CA Cancer J Clin. 2012;62(4):220–41. 10.3322/caac.21149. 22700443 10.3322/caac.21149

[6] Emery J, Boyle F, Jefford M, et al. Management of common clinical problems experienced by survivors of cancer. Lancet. 2022;399(10334):1537–50. 10.1016/S0140-6736(22)00242-2. 35430021 10.1016/S0140-6736(22)00242-2

[7] Oerlemans S, de Ligt K, Velthuis MJ, Siesling S, van der Poll-Franse LV, Ezendam NPM. (Over) leven met en na kanker: patiënten ervaren langdurige gevolgen van kanker en de behandeling. Ned Tijdschr Oncol. 2020;17(2):49–57.

[8] Porro B, Michelotti A, Hay JL, et al. Quality of life, fatigue and changes therein as predictors of return to work during breast cancer treatment. Scand J Caring Sci. 2019;33(2):467–77. 10.1111/scs.12646. 30664270 10.1111/scs.12646

[9] Lewis J, Mackenzie L. Cognitive changes after breast cancer: a scoping review to identify problems encountered by women when returning to work. Disabil Rehabil. 2021;44(18):5310–28. 10.1080/09638288.2021.1919216. 33974469 10.1080/09638288.2021.1919216

[10] Lilliehorn S, Hamberg K, Kero A, Salander P. Meaning of work and the returning process after breast cancer: a longitudinal study of 56 women. Scand J Caring Sci. 2012;27(2):267–74. 10.1111/j.1471-6712.2012.01026.x. 22671712 10.1111/j.1471-6712.2012.01026.x

[11] Fong DY, Ho JW, Hui BP, et al. Physical activity for cancer survivors: meta-analysis of randomised controlled trials. BMJ. 2012;344(jan30 5):e70. 10.1136/bmj.e70. 22294757 10.1136/bmj.e70 PMC3269661

[12] Cormie P, Zopf EM, Zhang X, Schmitz KH. The impact of exercise on cancer mortality, recurrence, and treatment-related adverse effects. Epidemiol Rev. 2017;39(1):71–92. 10.1093/epirev/mxx007. 28453622 10.1093/epirev/mxx007

[13] Wilson TN, Trainor D, Campbell KL, et al. Effectiveness of physical activity interventions on return to work after a cancer diagnosis: A systematic review and meta-analysis. J Occup Rehabil [Preprint]. 2022. 10.1007/s10926-022-10052-9. 35779184 10.1007/s10926-022-10052-9 PMC10025244

[14] Smits A, Lopes A, Derks M, et al. The effect of lifestyle interventions on the quality of life of gynaecological cancer survivors. Gynecol Oncol. 2015;139(3):546–52. 10.1016/j.ygyno.2015.10.002. 26441008 10.1016/j.ygyno.2015.10.002

[15] Kudre D, de Groef A, Geraerts I, et al. Multidisciplinary outpatient cancer rehabilitation can improve cancer patients’ physical and psychosocial status—a systematic review. Curr Oncol Rep. 2020;22(12). 10.1007/s11912-020-00979-8. 10.1007/s11912-020-00979-8 PMC7529622 33001322

[16] Butow PN, Sharpe L, Thewes B, et al. Randomized trial of conquerfear: a novel, theoretically based psychosocial intervention for fear of cancer recurrence. J Clin Oncol. 2017;35(36):4066–77. 10.1200/JCO.2017.73.1257. 29095681 10.1200/JCO.2017.73.1257

[17] Sung H, Hyun N, Leach CR, et al. Association of first primary cancer with risk of subsequent primary cancer among survivors of adult-onset cancers in the United States. JAMA. 2020;324(24):2521. 10.1001/jama.2020.23130. 33351041 10.1001/jama.2020.23130 PMC7756242

[18] Perez DG, Loprinzi CL, Ruddy KJ. Lifestyle factors can lead to multiple cancers over a lifetime—here we go again. JAMA Oncol. 2021;7(4):505. 10.1001/jamaoncol.2020.7360. 33351067 10.1001/jamaoncol.2020.7360

[19] Borsboom D. A network theory of mental disorders. World Psychiatry. 2017;16(1):5–13. 10.1002/wps.20375. 28127906 10.1002/wps.20375 PMC5269502

[20] Fried EI, van Borkulo CD, Cramer AO, et al. Mental disorders as networks of problems: A review of recent insights. Soc Psychiatry Psychiatr Epidemiol. 2016;52(1):1–10. 10.1007/s00127-016-1319-z. 27921134 10.1007/s00127-016-1319-z PMC5226976

[21] Jefford M, Howell D, Li J, et al. Improved models of care for cancer survivors. Lancet. 2022;399(10334):1551–60. 10.1016/S0140-6736(22)00306-3. 35430022 10.1016/S0140-6736(22)00306-3 PMC9009839

[22] NHG. Standpunt oncologische zorg in De Huisartsenpraktijk. Available at: https://www.nhg.org/themas/publicaties/nhg-standpunt-oncologische-zorg-de-huisartsenpraktijk. Accessed 15 Dec 2022.

[23] Beumeler LFE, Blom EE, Schröder CD, van der Graaf WTA. Evaluation of a lifestyle intervention program in primary care on physical and mental health and quality of life of cancer survivors: A pilot study. Eur J Integr Med. 2018;23:1–5. 10.1016/j.eujim.2018.08.007.

[24] Huang HH, Stubbs B, Chen LJ, et al. The effect of physical activity on sleep disturbance in various populations: a scoping review of randomized clinical trials. Int J Behav Nutr Phys Act. 2023;20(1):44. 37069626 10.1186/s12966-023-01449-7 PMC10107572

[25] Andersen AH, Vinther A, Poulsen LL, Mellemgaard A. A modified exercise protocol may promote continuance of exercise after the intervention in lung cancer patients—a pragmatic uncontrolled trial. Support Care Cancer. 2013;21:2247–53. 10.1007/s00520-013-1781-z. 23508894 10.1007/s00520-013-1781-z

[26] Thomsen S, Kristensen GDW, Jensen NWH, Agergaard S. Maintaining changes in physical activity among type 2 diabetics–a systematic review of rehabilitation interventions. Scand J Med Sci Sports. 2021;31(8):1582–91. 33735484 10.1111/sms.13951

[27] Fayers P, Bottomley A. Quality of life research within the EORTC—the EORTC QLQ-C30. Eur J Cancer. 2002;38:125–33. 10.1016/S0959-8049(01)00448-8. 10.1016/s0959-8049(01)00448-8 11858978

[28] Smets EMA, Garssen B, Bonke B, De Haes JC. The multidimensional fatigue inventory (MFI) psychometric qualities of an instrument to assess fatigue. J Psychosom Res. 1995;39(3):315–25. 10.1016/0022-3999(94)00125-O. 7636775 10.1016/0022-3999(94)00125-o

[29] De Beurs E, Van Dyck R, Marquenie LA, Lange A, Blonk RWB. De DASS: Een vragenlijst voor het meten van depressie, angst en stress. Gedragstherapie. 2001;34(1):35–53.

[30] Lovibond PF, Lovibond SH. The structure of negative emotional states: comparison of the depression anxiety stress scales (DASS) with the Beck depression and anxiety inventories. Behav Res Ther. 1995;33(3):335–43. 10.1016/0005-7967(94)00075-U. 7726811 10.1016/0005-7967(94)00075-u

[31] Abdel-Khalek AM. Measuring happiness with a single-item scale. Soc Behav Pers. 2006;34(2):139–50. 10.2224/sbp.2006.34.2.139.

[32] Ahlstrom L, Grimby-Ekman A, Hagberg M, Dellve L. The work ability index and single-item question: associations with sick leave, symptoms, and health – a prospective study of women on long-term sick leave. Scand J Work Environ Health. 2010;36(5):404–12. 10.5271/sjweh.2917. 20372766 10.5271/sjweh.2917

[33] Dorland HF, Abrahamsen JF, Thorsen L, et al. Work functioning trajectories in cancer patients: results from the longitudinal work life after cancer (Wolica) study. Int J Cancer. 2017;141(9):1751–62. 10.1002/ijc.30876. 28681478 10.1002/ijc.30876

[34] Cohen J. A power primer. Psychol Bull. 1992;112(1):155–9. 10.1037/0033-2909.112.1.155. 19565683 10.1037//0033-2909.112.1.155

[35] Cima RFF, Andersson G, Schmidt CJ, et al. Specialised treatment based on cognitive behaviour therapy versus usual care for tinnitus: A randomised controlled trial. Lancet. 2012;379(9830):1951–9. 10.1016/S0140-6736(12)60469-3. 22633033 10.1016/S0140-6736(12)60469-3

[36] Spark LC, Reeves MM, Fjeldsoe BS, Eakin EG. Physical activity and/or dietary interventions in breast cancer survivors: a systematic review of the maintenance of outcomes. J Cancer Surviv. 2012;7(1):74–82. 10.1007/s11764-012-0246-6. 23179496 10.1007/s11764-012-0246-6

[37] Leensen MC, Groeneveld IF, de Boer AGEM, et al. Return to work of cancer patients after a multidisciplinary intervention including occupational counselling and physical exercise: A prospective study in the Netherlands. BMJ Open. 2017;7(6):e014746. 10.1136/bmjopen-2016-014746. 28619770 10.1136/bmjopen-2016-014746 PMC5623345

[38] Pistelli M, Scartozzi M, Ballatore Z, et al. The impact of lifestyle interventions in high-risk early breast cancer patients: a modeling approach from a single institution experience. Cancers. 2021;13(21):5539. 10.3390/cancers13215539. 34771702 10.3390/cancers13215539 PMC8583345

[39] Vagnini D, Maruotti A, Pagliuca F, et al. Home-based lifestyle intervention for breast cancer survivors: a surprising improvement in the quality of life during the first year of COVID-19 pandemic. PLoS ONE. 2024;19(1):e0296163. 10.1371/journal.pone.0296163. 38165970 10.1371/journal.pone.0296163 PMC10760703

[40] Zikos E, Coens C, Quinten C, et al. The added value of analyzing pooled health-related quality of life data: a review of the EORTC PROBE initiative. J Natl Cancer Inst. 2015. 10.1093/jnci/djv391. 26714759 10.1093/jnci/djv391

[41] Giesinger JM, Kieffer JM, Fayers PM, et al. Thresholds for clinical importance were established to improve interpretation of the EORTC QLQ-C30 in clinical practice and research. J Clin Epidemiol. 2020;118:1–8. 10.1016/j.jclinepi.2019.10.003. 31639445 10.1016/j.jclinepi.2019.10.003

[42] de Boer AGEM, Taskila T, Tamminga SJ, et al. Interventions to enhance return-to-work for cancer patients. Cochrane Database Syst Rev. 2015;2017(7). 10.1002/14651858.CD007569.pub3. 10.1002/14651858.CD007569.pub3 PMC6483290 26405010

[43] Berglund G, Bolund C, Gustafsson UL, Sjödén P-O. Oneyear follow-up of the ‘Starting again’ group rehabilitation programme for cancer patients. Eur J Cancer. 1994;30A(12):1744–51. 7880598 10.1016/0959-8049(94)00330-8

[44] Maguire P, Brooke M, Tait A, Thomas C, Sellwood R. The effect of counselling on physical disability and social recovery aMer mastectomy. Clin Oncol. 1983;9(4):319–24. 6362943

[45] Burgio KL, Goode PS, Urban DA, Umlauf MG, Locher JL, Bueschen A, et al. Preoperative biofeedback assisted behavioral training to decrease post-prostatectomy incontinence: a randomized, controlled study. J Urol. 2006;175(1):196–201. 16406909 10.1016/S0022-5347(05)00047-9

[46] Hubbard G, Gray NM, Ayansina D, Evans JM, Kyle RG. Case management vocational rehabilitation for women with breast cancer after surgery: a feasibility study incorporating a pilot randomised controlled trial. Trials. 2013;14:175. 23768153 10.1186/1745-6215-14-175 PMC3698180

[47] Tamminga SJ, Verbeek JH, Bos MM, Fons G, Kitzen JJ, Plaisier PW, et al. Effectiveness of a hospital-based work support intervention for female cancer patients - a multi-centre randomised controlled trial. PLoS ONE. 2013;8(5):e63271. 23717406 10.1371/journal.pone.0063271 PMC3661555

[48] Schillemans C, Castelein S, de Vreede KL, Hoenders HJR, Booij SH. Assessing physical activity and sedentary behavior in people with mental illnesses: do actigraphy and daily self-report measures agree? Ment Health Phys Act. 2025;25:100699.

