# Supplementary material for: The Transdiagnostic Oncology Program (TOP): a multidomain lifestyle intervention to improve the quality of life of cancer survivors - a before-and-after pilot study in primary care
Source: BMC Cancer. 2025 Nov 10;25:1745. doi: 10.1186/s12885-025-15063-2 (PMC12604275; doi:10.1186/s12885-025-15063-2)
Supplement: Supplementary file 1 — Supplementary Material 1: Table S1. Summary of intervention components. [file 12885_2025_15063_MOESM1_ESM.docx]

**Table S1. Summary of intervention components.**

| **Component** | **Frequency** | **Duration** | **Mode** |
| --- | --- | --- | --- |
| Physical activity | 1-3 months: 2x a week  4-6 months: 1x a week | 1 hour | Group sessions, provided by physiotherapist. 25 minutes of cardio, 35 minutes of muscle strength training |
| Mind-body therapy | 1-6 months: 1x a week | 1 hour | Group sessions, provided by mindfulness trainer and hatha yoga trainer. Body scan, breathing exercise based on MBCT, Hata yoga exercises |
| Nutrition | 1 informational meeting, 2 personal appointments. More session can be scheduled, depending on personal need | 30 minutes | 1 group session, provided by dietician.  Thereafter, in person (face-to-face), provided by dietician. |
| Sleep hygiene | 1 informational session | 1 hour | Group session, provided by family doctor and physiotherapist. |
| Psychological therapy | 4 appointments with the family doctor. Depending on personal need, referral to psycho-oncology institute | 30 minutes | In person (face-to-face), provided by the family doctor.  Thereafter, if needed, in person (face-to-face) appointments with psychologist. |

Note. MBCT = Mindfulness-based cognitive therapy.
