# Supplementary material for: The Transdiagnostic Oncology Program (TOP): a multidomain lifestyle intervention to improve the quality of life of cancer survivors - a before-and-after pilot study in primary care
Source: BMC Cancer. 2025 Nov 10;25:1745. doi: 10.1186/s12885-025-15063-2 (PMC12604275; doi:10.1186/s12885-025-15063-2)
Supplement: Supplementary file 2 — Supplementary Material 2: Table S2. An overview of the measurements. [file 12885_2025_15063_MOESM2_ESM.docx]

|  | | | | | |
| --- | --- | --- | --- | --- | --- |
| Measurements |  | **T_-1_** | **T_0_^a^ (Baseline)** | **T_1_** | **T_2_^a^** |
| Outcome measurements | Instruments | -1 mth | 0 mth | 6 mth | 12 mth |
| *Primary outcomes* |  |  |  |  |  |
| Quality of life (QoL) | EORTC QLQ-C30 | FD | FD | FD | FD |
| *Secondary outcomes* | | | |  |  |
| Pain | NPRS |  | PT | PT | PT |
| Fatigue | MVI-20 | FD | FD | FD | FD |
| Mental well-being | DASS-21 | FD | FD | FD | FD |
| Happiness | HI | FD | FD | FD | FD |
| Return to work & work-related functioning | RTW & work functioning questionnaire including: - sociodemographic factors and employment status - WAI - contextual questions about work accommodations |  | FD | FD | FD |
| Feasibility and satisfaction | Evaluation form |  |  |  | FD |
| *_Note:_* _The questionnaires were collected by the family doctor (FD) or the physical therapists (PT)._ ^a^_control group included, except for the grip strength and the feasibility measurements._ | | | | | |

**Table S2. An overview of the measurements.**
