# Supplementary material for: The Transdiagnostic Oncology Program (TOP): a multidomain lifestyle intervention to improve the quality of life of cancer survivors - a before-and-after pilot study in primary care
Source: BMC Cancer. 2025 Nov 10;25:1745. doi: 10.1186/s12885-025-15063-2 (PMC12604275; doi:10.1186/s12885-025-15063-2)
Supplement: Supplementary file 3 — Supplementary Material 3: Text S1. Evaluation form. [file 12885_2025_15063_MOESM3_ESM.docx]

**Evaluation Form**

**Content and format of the training**

**What overall grade would you give the content of the entire training?**

☐ 1 ☐ 2 ☐ 3 ☐ 4 ☐ 5 ☐ 6 ☐ 7 ☐ 8 ☐ 9 ☐ 10

**What did you think of the duration of the TOP program; 1 year?**

☐ Too short ☐ Just right ☐ Too long

**What did you think of the overall burden of the entire TOP program?**

☐ Too light ☐ Adequate ☐ Too heavy

**Was it feasible for you to follow the entire TOP program?**

☐ Not at all ☐ Somewhat ☐ Readily feasible ☐ Difficult to manage

**If applicable: why was it difficult and/or not feasible to follow the entire TOP program?**

☐ A) difficult in terms of planning/schedule ☐ B) difficult in terms of energy ☐ C) difficult in terms of motivation ☐ D) difficult in terms of transportation ☐ E) difficult due to recurrence of illness ☐ F) other, namely …

__________________________________________________________________________________________

**What did you think of the intensity of the PT (physical therapy) part of the TOP program?**

☐ Too light ☐ Adequate ☐ Too heavy

**What did you think of the 1-hour training time for the PT part of the TOP program?**

☐ Too short ☐ Just right ☐ Too long

**What did you think of the frequency of the PT training (twice per week for 3 months and once per week for 3 months)?**

☐ Too often ☐ Just right ☐ Too little

**How did you find training in a group setting with the physical therapist?**

☐ Unpleasant ☐ Neutral ☐ Pleasant

If applicable, please explain:

__________________________________________________________________________________________

__________________________________________________________________________________________

__________________________________________________________________________________________

**What did you think of the duration of the RELAXATION component of the TOP program (45 minutes per session)?**

☐ Too short ☐ Just right ☐ Too long

**What did you think of the frequency of the RELAXATION component (once per week for 3 months)?**

☐ Too short ☐ Just right ☐ Too long

**How did you find doing the relaxation exercises in a group setting?**

☐ Unpleasant ☐ Neutral ☐ Pleasant

If applicable, please explain:

__________________________________________________________________________________________

__________________________________________________________________________________________

**What did you think of the time spent with the Dietitian (30 minutes per visit)?**

☐ Too short ☐ Just right ☐ Too long

**How many appointments did you have with the Dietitian?**

☐ 1 ☐ 2 ☐ 3 ☐ 4 ☐ 5 ☐ 6 ☐ 7 ☐ 8 ☐ 9 ☐ 10

**How did you find the number of contacts with the Dietitian?**

☐ Too few ☐ Just right ☐ Too many

**What did you think of the time spent with the primary care physician (30 hours per visit [sic])?**

☐ Too short ☐ Just right ☐ Too long

**What did you think of the frequency of visits with the primary care physician (4× per year)?**

☐ Too infrequent ☐ Just right ☐ Too frequent

**Did you find it valuable to receive a combined offering (exercise, relaxation, dietitian, and primary care physician)? (1 = not at all / 10 = very much)**

☐ 1 ☐ 2 ☐ 3 ☐ 4 ☐ 5 ☐ 6 ☐ 7 ☐ 8 ☐ 9 ☐ 10

Could you elaborate?

__________________________________________________________________________________________

__________________________________________________________________________________________

**How did you find training on your own after the first half-year with guidance?**

☐ Unpleasant ☐ Neutral ☐ Pleasant

If applicable, please explain:

__________________________________________________________________________________________

__________________________________________________________________________________________

**Did the TOP program improve your quality of life? (1 = not at all / 10 = very much)**

☐ 1 ☐ 2 ☐ 3 ☐ 4 ☐ 5 ☐ 6 ☐ 7 ☐ 8 ☐ 9 ☐ 10

Could you elaborate?

__________________________________________________________________________________________

__________________________________________________________________________________________

**Did you find it burdensome to complete the various questionnaires? (1 = not at all / 10 = extremely)**

☐ 1 ☐ 2 ☐ 3 ☐ 4 ☐ 5 ☐ 6 ☐ 7 ☐ 8 ☐ 9 ☐ 10

Could you elaborate?

__________________________________________________________________________________________

__________________________________________________________________________________________

**Were there topics or exercises you missed in the TOP program?**

☐ No ☐ Yes, namely …

__________________________________________________________________________________________

**Would you recommend the TOP program to someone else?**

☐ No ☐ Maybe ☐ Yes

Could you elaborate?

__________________________________________________________________________________________

**Do you have any additional comments about the content or format of the TOP program?**

__________________________________________________________________________________________

__________________________________________________________________________________________

__________________________________________________________________________________________

**Exercise and outcomes**

The training was designed with several goals in mind. We would like to know the extent to which you agree with the following statements. Please check the box that matches your choice.

Scale: 1 = Strongly disagree, 2 = Somewhat disagree, 3 = Neither agree nor disagree, 4 = Somewhat agree, 5 = Strongly agree

I have made a structural change to my physical activity/exercise compared with a year ago.

☐ 1 ☐ 2 ☐ 3 ☐ 4 ☐ 5

If yes, what did you change?

__________________________________________________________________________________________

I have made a structural change to my STRESS level compared with a year ago.

☐ 1 ☐ 2 ☐ 3 ☐ 4 ☐ 5

If yes, what did you change?

__________________________________________________________________________________________

I have made a structural change to my eating pattern compared with a year ago.

☐ 1 ☐ 2 ☐ 3 ☐ 4 ☐ 5

If yes, what did you change?

__________________________________________________________________________________________

I have started to look differently at the illness I have (or had).

☐ 1 ☐ 2 ☐ 3 ☐ 4 ☐ 5

The lifestyle changes I built up during the first half-year I have been able to maintain up to now.

☐ 1 ☐ 2 ☐ 3 ☐ 4 ☐ 5

I received enough practical tools to get started on my own with Exercise.

☐ 1 ☐ 2 ☐ 3 ☐ 4 ☐ 5

I received enough practical tools to get started on my own with Nutrition.

☐ 1 ☐ 2 ☐ 3 ☐ 4 ☐ 5

I received enough practical tools to get started on my own with RELAXATION exercises.

☐ 1 ☐ 2 ☐ 3 ☐ 4 ☐ 5

The various projects from the participant binder added value to my personal goals within the TOP program.

☐ 1 ☐ 2 ☐ 3 ☐ 4 ☐ 5

**26. Have you experienced any adverse effects from the TOP program?**

__________________________________________________________________________________________

__________________________________________________________________________________________

**27. What are your plans regarding exercise, relaxation, and nutrition after completing the TOP program?**

__________________________________________________________________________________________

__________________________________________________________________________________________

How do you plan to approach this?

__________________________________________________________________________________________

__________________________________________________________________________________________

**The trainers**

If you could give the following providers a grade, what would you give them, considering their role as providers in the training?

**28. How satisfied are you in general with the PT guidance? (please check)**

☐ 1 ☐ 2 ☐ 3 ☐ 4 ☐ 5 ☐ 6 ☐ 7 ☐ 8 ☐ 9 ☐ 10

Could you elaborate?

__________________________________________________________________________________________

__________________________________________________________________________________________

**29. How satisfied are you with the RELAXATION guidance? (please check)**

☐ 1 ☐ 2 ☐ 3 ☐ 4 ☐ 5 ☐ 6 ☐ 7 ☐ 8 ☐ 9 ☐ 10

Could you elaborate?

__________________________________________________________________________________________

__________________________________________________________________________________________

**30. How satisfied are you with the Dietitian’s guidance? (please check)**

☐ 1 ☐ 2 ☐ 3 ☐ 4 ☐ 5 ☐ 6 ☐ 7 ☐ 8 ☐ 9 ☐ 10

Could you elaborate?

__________________________________________________________________________________________

__________________________________________________________________________________________

**31. How satisfied are you with the primary care physician’s guidance (Elkana Waarsenburg)? (please check)**

☐ 1 ☐ 2 ☐ 3 ☐ 4 ☐ 5 ☐ 6 ☐ 7 ☐ 8 ☐ 9 ☐ 10

Could you elaborate?

__________________________________________________________________________________________

__________________________________________________________________________________________

**32. If applicable, how satisfied are you with the guidance at the Behouden Huys? (please check)**

☐ 1 ☐ 2 ☐ 3 ☐ 4 ☐ 5 ☐ 6 ☐ 7 ☐ 8 ☐ 9 ☐ 10

Could you elaborate?

__________________________________________________________________________________________

__________________________________________________________________________________________

**Practical organization**

33. For each topic below, please indicate how satisfied you were: 1 = Very dissatisfied, 2 = Dissatisfied, 3 = Neutral, 4 = Satisfied, 5 = Very satisfied

| Topic | Select one: 1–5 |
| --- | --- |
| The course materials | ☐ 1 ☐ 2 ☐ 3 ☐ 4 ☐ 5 |
| The information provided beforehand | ☐ 1 ☐ 2 ☐ 3 ☐ 4 ☐ 5 |
| The information evening regarding TOP participation (Nov 2018) | ☐ 1 ☐ 2 ☐ 3 ☐ 4 ☐ 5 |
| The information evening regarding TOP background and nutrition (Feb 2019) | ☐ 1 ☐ 2 ☐ 3 ☐ 4 ☐ 5 |
| The information evening about sleep (July 2019) | ☐ 1 ☐ 2 ☐ 3 ☐ 4 ☐ 5 |

**34. Do you have any general additions or suggestions for improvement for this training?**

__________________________________________________________________________________________

__________________________________________________________________________________________

__________________________________________________________________________________________
