# Supplementary material for: The Transdiagnostic Oncology Program (TOP): a multidomain lifestyle intervention to improve the quality of life of cancer survivors - a before-and-after pilot study in primary care
Source: BMC Cancer. 2025 Nov 10;25:1745. doi: 10.1186/s12885-025-15063-2 (PMC12604275; doi:10.1186/s12885-025-15063-2)
Supplement: Supplementary file 4 — Supplementary Material 4: Text S2. Specification of the multilevel models. [file 12885_2025_15063_MOESM4_ESM.docx]

**Specifications of the multilevel models**

For the models that assessed time*group interactions, we assumed a compound symmetry (CS) covariance matrix for the repeated outcome measures, and it also resulted in best model fit in almost all models (according to the likelihood ratio test).

For the models that assessed effects over time within the intervention group, we assumed first-order autoregressive (AR1) covariance matrix for the repeated outcome measures based on theory and indeed found this matrix to be the best fitting for most models (according to the likelihood ratio test).

Restricted maximum likelihood estimation was used to find the best fit for the random effects and covariance matrix. Maximum likelihood estimation was subsequently used to estimate and interpret the (unbiased) fixed effects.
