# Supplementary material for: The Transdiagnostic Oncology Program (TOP): a multidomain lifestyle intervention to improve the quality of life of cancer survivors - a before-and-after pilot study in primary care
Source: BMC Cancer. 2025 Nov 10;25:1745. doi: 10.1186/s12885-025-15063-2 (PMC12604275; doi:10.1186/s12885-025-15063-2)
Supplement: Supplementary file 5 — Supplementary Material 5: Table S3. All results of the evaluation questionnaire. [file 12885_2025_15063_MOESM5_ESM.docx]

**Table S3. All results of the evaluation questionnaire**

| **Duration TOP** | | | | | |
| --- | --- | --- | --- | --- | --- |
|  | | Frequency | Percent | Valid Percent | Cumulative Percent |
| Valid | Too short | 2 | 14,3 | 15,4 | 15,4 |
|  | Exactly right | 11 | 78,6 | 84,6 | 100,0 |
|  | Total | 13 | 92,9 | 100,0 |  |
| Missing | System | 1 | 7,1 |  |  |
| Total | | 14 | 100,0 |  |  |

| **Burden TOP** | | | | | |
| --- | --- | --- | --- | --- | --- |
|  | | Frequency | Percent | Valid Percent | Cumulative Percent |
| Valid | Exactly right | 13 | 92,9 | 100,0 | 100,0 |
| Missing | System | 1 | 7,1 |  |  |
| Total | | 14 | 100,0 |  |  |

| **Feasibility TOP** | | | | | |
| --- | --- | --- | --- | --- | --- |
|  | | Frequency | Percent | Valid Percent | Cumulative Percent |
| Valid | Somewhat | 2 | 14,3 | 15,4 | 15,4 |
|  | Good | 11 | 78,6 | 84,6 | 100,0 |
|  | Total | 13 | 92,9 | 100,0 |  |
| Missing | System | 1 | 7,1 |  |  |
| Total | | 14 | 100,0 |  |  |

| **Intensity TOP** | | | | | |
| --- | --- | --- | --- | --- | --- |
|  | | Frequency | Percent | Valid Percent | Cumulative Percent |
| Valid | Exactly right | 12 | 85,7 | 92,3 | 92,3 |
|  | Too high | 1 | 7,1 | 7,7 | 100,0 |
|  | Total | 13 | 92,9 | 100,0 |  |
| Missing | System | 1 | 7,1 |  |  |
| Total | | 14 | 100,0 |  |  |

| **Duration physiotherapy sessions** | | | | | |
| --- | --- | --- | --- | --- | --- |
|  | | Frequency | Percent | Valid Percent | Cumulative Percent |
| Valid |  | 1 | 7,1 | 7,1 | 7,1 |
|  | 1 | 2 | 14,3 | 14,3 | 21,4 |
|  | 1.5 | 1 | 7,1 | 7,1 | 28,6 |
|  | 2 | 10 | 71,4 | 71,4 | 100,0 |
|  | Total | 14 | 100,0 | 100,0 |  |

| **Frequency physiotherapy sessions** | | | | | |
| --- | --- | --- | --- | --- | --- |
|  | | Frequency | Percent | Valid Percent | Cumulative Percent |
| Valid | Too low | 1 | 7,1 | 7,7 | 7,7 |
|  | Exactly right | 8 | 57,1 | 61,5 | 69,2 |
|  | Too high | 4 | 28,6 | 30,8 | 100,0 |
|  | Total | 13 | 92,9 | 100,0 |  |
| Missing | System | 1 | 7,1 |  |  |
| Total | | 14 | 100,0 |  |  |

| **Group aspect physiotherapy sessions** | | | | | |
| --- | --- | --- | --- | --- | --- |
|  | | Frequency | Percent | Valid Percent | Cumulative Percent |
| Valid | Neutral | 5 | 35,7 | 38,5 | 38,5 |
|  | Pleasant | 8 | 57,1 | 61,5 | 100,0 |
|  | Total | 13 | 92,9 | 100,0 |  |
| Missing | System | 1 | 7,1 |  |  |
| Total | | 14 | 100,0 |  |  |

| **Duration relaxation sessions** | | | | | |
| --- | --- | --- | --- | --- | --- |
|  | | Frequency | Percent | Valid Percent | Cumulative Percent |
| Valid | Too short | 2 | 14,3 | 18,2 | 18,2 |
|  | Exactly right | 8 | 57,1 | 72,7 | 90,9 |
|  | Too long | 1 | 7,1 | 9,1 | 100,0 |
|  | Total | 11 | 78,6 | 100,0 |  |
| Missing | System | 3 | 21,4 |  |  |
| Total | | 14 | 100,0 |  |  |

| **Frequency relaxation sessions** | | | | | |
| --- | --- | --- | --- | --- | --- |
|  | | Frequency | Percent | Valid Percent | Cumulative Percent |
| Valid | Too low | 6 | 42,9 | 54,5 | 54,5 |
|  | Exactly right | 4 | 28,6 | 36,4 | 90,9 |
|  | Too high | 1 | 7,1 | 9,1 | 100,0 |
|  | Total | 11 | 78,6 | 100,0 |  |
| Missing | System | 3 | 21,4 |  |  |
| Total | | 14 | 100,0 |  |  |

| **Group aspect relaxation sessions** | | | | | |
| --- | --- | --- | --- | --- | --- |
|  | | Frequency | Percent | Valid Percent | Cumulative Percent |
| Valid | Upleasant | 2 | 14,3 | 18,2 | 18,2 |
|  | Neutral | 5 | 35,7 | 45,5 | 63,6 |
|  | Pleasant | 4 | 28,6 | 36,4 | 100,0 |
|  | Total | 11 | 78,6 | 100,0 |  |
| Missing | System | 3 | 21,4 |  |  |
| Total | | 14 | 100,0 |  |  |

| **Duration appointments nutritionist sessions** | | | | | |
| --- | --- | --- | --- | --- | --- |
|  | | Frequency | Percent | Valid Percent | Cumulative Percent |
| Valid | Exactly right | 11 | 78,6 | 84,6 | 84,6 |
|  | Too long | 2 | 14,3 | 15,4 | 100,0 |
|  | Total | 13 | 92,9 | 100,0 |  |
| Missing | System | 1 | 7,1 |  |  |
| Total | | 14 | 100,0 |  |  |

| **Number of appointments nutritionist sessions** | | | | | |
| --- | --- | --- | --- | --- | --- |
|  | | Frequency | Percent | Valid Percent | Cumulative Percent |
| Valid | 2 | 1 | 7,1 | 8,3 | 8,3 |
|  | 3 | 2 | 14,3 | 16,7 | 25,0 |
|  | 4 | 3 | 21,4 | 25,0 | 50,0 |
|  | 5 | 4 | 28,6 | 33,3 | 83,3 |
|  | 7 | 1 | 7,1 | 8,3 | 91,7 |
|  | 9 | 1 | 7,1 | 8,3 | 100,0 |
|  | Total | 12 | 85,7 | 100,0 |  |
| Missing | System | 2 | 14,3 |  |  |
| Total | | 14 | 100,0 |  |  |

| **Frequency nutritionist sessions** | | | | | |
| --- | --- | --- | --- | --- | --- |
|  | | Frequency | Percent | Valid Percent | Cumulative Percent |
| Valid | Exactly right | 11 | 78,6 | 84,6 | 84,6 |
|  | Too high | 2 | 14,3 | 15,4 | 100,0 |
|  | Total | 13 | 92,9 | 100,0 |  |
| Missing | System | 1 | 7,1 |  |  |
| Total | | 14 | 100,0 |  |  |

| **Duration appointments Family Doctor** | | | | | |
| --- | --- | --- | --- | --- | --- |
|  | | Frequency | Percent | Valid Percent | Cumulative Percent |
| Valid | Too short | 1 | 7,1 | 7,7 | 7,7 |
|  | Exactly right | 12 | 85,7 | 92,3 | 100,0 |
|  | Total | 13 | 92,9 | 100,0 |  |
| Missing | System | 1 | 7,1 |  |  |
| Total | | 14 | 100,0 |  |  |

| **Frequency appointments Family Doctor** | | | | | |
| --- | --- | --- | --- | --- | --- |
|  | | Frequency | Percent | Valid Percent | Cumulative Percent |
| Valid | Too low | 1 | 7,1 | 7,7 | 7,7 |
|  | Exactly right | 12 | 85,7 | 92,3 | 100,0 |
|  | Total | 13 | 92,9 | 100,0 |  |
| Missing | System | 1 | 7,1 |  |  |
| Total | | 14 | 100,0 |  |  |

| **Ease of transition to individual training** | | | | | |
| --- | --- | --- | --- | --- | --- |
|  | | Frequency | Percent | Valid Percent | Cumulative Percent |
| Valid | Upleasant | 2 | 14,3 | 15,4 | 15,4 |
|  | Neutral | 5 | 35,7 | 38,5 | 53,8 |
|  | Pleasant | 6 | 42,9 | 46,2 | 100,0 |
|  | Total | 13 | 92,9 | 100,0 |  |
| Missing | System | 1 | 7,1 |  |  |
| Total | | 14 | 100,0 |  |  |

| **Missing themes or exercises** | | | | | |
| --- | --- | --- | --- | --- | --- |
|  | | Frequency | Percent | Valid Percent | Cumulative Percent |
| Valid | No | 9 | 64,3 | 69,2 | 69,2 |
|  | Yes | 4 | 28,6 | 30,8 | 100,0 |
|  | Total | 13 | 92,9 | 100,0 |  |
| Missing | System | 1 | 7,1 |  |  |
| Total | | 14 | 100,0 |  |  |

| **Recommending TOP to others** | | | | | |
| --- | --- | --- | --- | --- | --- |
|  | | Frequency | Percent | Valid Percent | Cumulative Percent |
| Valid | Maybe | 1 | 7,1 | 7,7 | 7,7 |
|  | Yes | 12 | 85,7 | 92,3 | 100,0 |
|  | Total | 13 | 92,9 | 100,0 |  |
| Missing | System | 1 | 7,1 |  |  |
| Total | | 14 | 100,0 |  |  |

| **Structural change Exercise** | | | | | |
| --- | --- | --- | --- | --- | --- |
|  | | Frequency | Percent | Valid Percent | Cumulative Percent |
| Valid | Totally disagree | 2 | 14,3 | 15,4 | 15,4 |
|  | Neutral | 4 | 28,6 | 30,8 | 46,2 |
|  | Somewhat agree | 3 | 21,4 | 23,1 | 69,2 |
|  | Totally agree | 4 | 28,6 | 30,8 | 100,0 |
|  | Total | 13 | 92,9 | 100,0 |  |
| Missing | System | 1 | 7,1 |  |  |
| Total | | 14 | 100,0 |  |  |

| **Structural change Stress** | | | | | |
| --- | --- | --- | --- | --- | --- |
|  | | Frequency | Percent | Valid Percent | Cumulative Percent |
| Valid | Totally disagree | 2 | 14,3 | 15,4 | 15,4 |
|  | Somewhat disagree | 1 | 7,1 | 7,7 | 23,1 |
|  | Neutral | 4 | 28,6 | 30,8 | 53,8 |
|  | Somewhat agree | 3 | 21,4 | 23,1 | 76,9 |
|  | Totally agree | 3 | 21,4 | 23,1 | 100,0 |
|  | Total | 13 | 92,9 | 100,0 |  |
| Missing | System | 1 | 7,1 |  |  |
| Total | | 14 | 100,0 |  |  |

| **Structural change Nutrition** | | | | | |
| --- | --- | --- | --- | --- | --- |
|  | | Frequency | Percent | Valid Percent | Cumulative Percent |
| Valid | Somewhat disagree | 2 | 14,3 | 15,4 | 15,4 |
|  | Neutral | 3 | 21,4 | 23,1 | 38,5 |
|  | Somewhat agree | 6 | 42,9 | 46,2 | 84,6 |
|  | Totally agree | 2 | 14,3 | 15,4 | 100,0 |
|  | Total | 13 | 92,9 | 100,0 |  |
| Missing | System | 1 | 7,1 |  |  |
| Total | | 14 | 100,0 |  |  |

| **Gained different look at illness** | | | | | |
| --- | --- | --- | --- | --- | --- |
|  | | Frequency | Percent | Valid Percent | Cumulative Percent |
| Valid | Totally disagree | 2 | 14,3 | 15,4 | 15,4 |
|  | Somewhat disagree | 2 | 14,3 | 15,4 | 30,8 |
|  | Neutral | 3 | 21,4 | 23,1 | 53,8 |
|  | Somewhat agree | 4 | 28,6 | 30,8 | 84,6 |
|  | Totally agree | 2 | 14,3 | 15,4 | 100,0 |
|  | Total | 13 | 92,9 | 100,0 |  |
| Missing | System | 1 | 7,1 |  |  |
| Total | | 14 | 100,0 |  |  |

| **Durable changes lifestyle** | | | | | |
| --- | --- | --- | --- | --- | --- |
|  | | Frequency | Percent | Valid Percent | Cumulative Percent |
| Valid | Totally disagree | 1 | 7,1 | 7,7 | 7,7 |
|  | Neutral | 3 | 21,4 | 23,1 | 30,8 |
|  | Somewhat agree | 4 | 28,6 | 30,8 | 61,5 |
|  | Totally agree | 5 | 35,7 | 38,5 | 100,0 |
|  | Total | 13 | 92,9 | 100,0 |  |
| Missing | System | 1 | 7,1 |  |  |
| Total | | 14 | 100,0 |  |  |

| **Sufficient tools exercise** | | | | | |
| --- | --- | --- | --- | --- | --- |
|  | | Frequency | Percent | Valid Percent | Cumulative Percent |
| Valid | Somewhat disagree | 1 | 7,1 | 7,7 | 7,7 |
|  | Somewhat agree | 5 | 35,7 | 38,5 | 46,2 |
|  | Totally agree | 7 | 50,0 | 53,8 | 100,0 |
|  | Total | 13 | 92,9 | 100,0 |  |
| Missing | System | 1 | 7,1 |  |  |
| Total | | 14 | 100,0 |  |  |

| **Sufficient tools nutrition** | | | | | |
| --- | --- | --- | --- | --- | --- |
|  | | Frequency | Percent | Valid Percent | Cumulative Percent |
| Valid | Totally disagree | 1 | 7,1 | 7,7 | 7,7 |
|  | Neutral | 1 | 7,1 | 7,7 | 15,4 |
|  | Somewhat agree | 4 | 28,6 | 30,8 | 46,2 |
|  | Totally agree | 7 | 50,0 | 53,8 | 100,0 |
|  | Total | 13 | 92,9 | 100,0 |  |
| Missing | System | 1 | 7,1 |  |  |
| Total | | 14 | 100,0 |  |  |

| **Sufficient tools relaxation** | | | | | |
| --- | --- | --- | --- | --- | --- |
|  | | Frequency | Percent | Valid Percent | Cumulative Percent |
| Valid | Totally disagree | 1 | 7,1 | 7,7 | 7,7 |
|  | Somewhat disagree | 1 | 7,1 | 7,7 | 15,4 |
|  | Neutral | 3 | 21,4 | 23,1 | 38,5 |
|  | Somewhat agree | 3 | 21,4 | 23,1 | 61,5 |
|  | Totally agree | 5 | 35,7 | 38,5 | 100,0 |
|  | Total | 13 | 92,9 | 100,0 |  |
| Missing | System | 1 | 7,1 |  |  |
| Total | | 14 | 100,0 |  |  |

| **Descriptive Statistics** | | | | | |
| --- | --- | --- | --- | --- | --- |
|  | N | Minimum | Maximum | Mean | Std. Deviation |
| Overall mark | 13 | 7 | 9 | 8,15 | ,801 |
| Number of appointments nutritionist sessions | 12 | 2 | 9 | 4,67 | 1,875 |
| Added value multidomain intervention | 13 | 7 | 10 | 9,23 | 1,092 |
| Quality of life improvement | 13 | 7 | 10 | 8,38 | 1,121 |
| Burden questionnaires | 13 | 1 | 7 | 3,85 | 2,641 |
| Valid N (listwise) | 12 |  |  |  |  |
