# Supplementary material for: The Transdiagnostic Oncology Program (TOP): a multidomain lifestyle intervention to improve the quality of life of cancer survivors - a before-and-after pilot study in primary care
Source: BMC Cancer. 2025 Nov 10;25:1745. doi: 10.1186/s12885-025-15063-2 (PMC12604275; doi:10.1186/s12885-025-15063-2)
Supplement: Supplementary file 6 — Supplementary Material 6: Table S4. Posthoc Intention-to-treat multilevel analyses to compare the change in outcome variables between the intervention and control group. [file 12885_2025_15063_MOESM6_ESM.docx]

| **Table S4. Posthoc Intention-to-treat multilevel analyses to compare the change in outcome variables between the intervention and control group.** | | | | | | |
| --- | --- | --- | --- | --- | --- | --- |
| **Primary outcomes** | | **B** | **95% C.I** | | | **P -value** |
| *Quality of life (EORTC QLQ-C30)* | | | *Lower* | *Upper* | |  |
| Global health status | Baseline | 72.54 | 66.28 | 78.82 | | .000* |
|  | Time | 3.89 | -4.54 | 12.33 | | .352 |
|  | Interaction | 2.96 | -7.75 | 13.68 | | .577 |
| Functioning scale | | | | | | |
| Physical functioning | Baseline | 85.69 | 79.55 | 91.82 | | .000* |
|  | Time | -1.51 | -7.14 | 4.12 | | .588 |
|  | Interaction | 8.20 | 0.89 | 15.50 | | .029* |
| Role functioning ^a^ | Baseline | 77.45 | 70.25 | 84.65 | | .000* |
|  | Time | 3.49 | -9.91 | 16.89 | | .601 |
|  | Interaction | 3.28 | -12.80 | 19.35 | | .684 |
| Emotional functioning | Baseline | 76.47 | 68.88 | 84.06 | | .000* |
|  | Time | 1.47 | -7.84 | 10.77 | | .748 |
|  | Interaction | -0.33 | -12.23 | 11.58 | | .956 |
| Cognitive functioning | Baseline | 79.41 | 73.31 | 85.52 | | .000* |
|  | Time | 4.57 | -4.38 | 13.51 | | .306 |
|  | Interaction | -3.52 | -14.89 | 7.65 | | .520 |
| Social functioning | Baseline | 81.86 | 75.39 | 88.34 | | .000* |
|  | Time | 7.70 | -3.45 | 18.86 | | .170 |
|  | Interaction | 7.76 | -5.917 | 21.44 | | .259 |
| Symptom scale | | | | | | |
| Fatigue | Baseline | 33.99 | 26.62 | 41.35 | | .000* |
|  | Time | -1.12 | -9.88 | 7.65 | | .796 |
|  | Interaction | -13.92 | -25.16 | -9.88 | | .017* |
| Pain | Baseline | 15.20 | 7.51 | 22.89 | | .000* |
|  | Time | 5.21 | -4.97 | 15.40 | | .305 |
|  | Interaction | -8.59 | -21.56 | 4.38 | | .187 |
| **Secondary outcomes** | | | | | | |
| *Fatigue (Multidimensional Fatigue Inventory)* | | | | | | |
| General Fatigue | Baseline | 12.64 | 10.86 | 14.41 | | .000* |
|  | Time | -0.09 | -2.30 | 2.11 | | .931 |
|  | Interaction | -2.42 | -5.23 | 0.40 | | .090 |
| Physical Fatigue | Baseline | 11.27 | 9.43 | 13.12 | | .000* |
|  | Time | 0.58 | -1.33 | 2.49 | | .537 |
|  | Interaction | -2.64 | -5.10 | -0.17 | | .037* |
| Reduced Activity | Baseline | 11.24 | 9.63 | 12.86 | | .000* |
|  | Time | -0.86 | -2.63 | 0.90 | | .326 |
|  | Interaction | -0.95 | -3.22 | 1.33 | | .403 |
| Reduced Motivation | Baseline | 9.24 | 7.76 | 10.72 | | .000* |
|  | Time | -0.29 | -1.95 | 1.36 | | .720 |
|  | Interaction | -1.22 | -3.35 | 0.90 | | .250 |
| Mental Fatigue | Baseline | 10.49 | 8.84 | 12.13 | | .000* |
|  | Time | 0.33 | -1.39 | 2.06 | | .694 |
|  | Interaction | -1.36 | -3.58 | 0.86 | | .221 |
| *Mental symptoms (Depression, Anxiety and Stress Scale)* | | | | | | |
| Depression ^a^ | Baseline | 4.55 | 2.59 | 6.50 | | .000* |
|  | Time | -0.61 | -2.79 | 1.56 | | .566 |
|  | Interaction | -0.03 | -2.82 | 2.77 | | .985 |
| Anxiety | Baseline | 4.18 | 2.47 | 5.89 | | .000* |
|  | Time | -0.03 | -1.24 | 1.18 | | .958 |
|  | Interaction | -1.89 | -3.47 | -0.32 | | .021* |
| Stress | Baseline | 7.27 | 4.56 | 9.98 | | .000* |
|  | Time | 0.14 | -2.77 | 3.05 | | .923 |
|  | Interaction | -1.72 | -5.48 | 2.03 | | .355 |
| *Happiness (Happiness Index)* | |  |  |  |  |  |
| Happiness ^a^ | Baseline | 6.56 | 5.67 | 7.46 | | .000* |
|  | Time | 0.23 | -0.69 | 1.14 | | .614 |
|  | Interaction | -0.32 | -1.23 | 1.16 | | .956 |
| *Work-related functioning (Work Ability Index)* | | | | | | |
| Work ability | Baseline | 6.56 | 5.67 | 7.46 | | .000* |
|  | Time | 0.23 | -0.69 | 1.14 | | .614 |
|  | Interaction | -0.03 | -1.23 | 1.16 | | .956 |
| *Significant p-value.  ^a^ If the residuals were skewed, a bootstrapped analysis was performed. The confidence interval and p-value are from the bootstrapped analysis. | | | | | | |
