# Supplementary material for: The Transdiagnostic Oncology Program (TOP): a multidomain lifestyle intervention to improve the quality of life of cancer survivors - a before-and-after pilot study in primary care
Source: BMC Cancer. 2025 Nov 10;25:1745. doi: 10.1186/s12885-025-15063-2 (PMC12604275; doi:10.1186/s12885-025-15063-2)
Supplement: Supplementary file 7 — Supplementary Material 7: Figure S1. Work-related functioning. [file 12885_2025_15063_MOESM7_ESM.docx]

**Figure S1. Change in working hours.**

^a^

*^Note^*^. The change in the number of working hours compared to the number of working hours before the diagnosis of cancer for both groups from baseline (T0) to post-intervention (T2).^

Figure S1 displays the change in the number of working hours at baseline and post-intervention, as compared to the number of working hours before the diagnosis All participants that worked before diagnoses (4 of control; 6 of intervention) worked equal working hours (2 of control; 0 of intervention) or less at baseline (range -40 till -0). The intervention group worked on average 19 hours less (range -5 till -40) and the control group 5 hours less (range 0 till -16), at baseline. All but one individual in the intervention group increased their working hours from baseline to post-intervention, while all individuals in the control group stayed stable. Two participants of the intervention group regained the same workhours as before (14-16 hours), the other two almost (from -40 to -4 and -24 to -5) and one worked more hours. The control group also did not change their function or work activities. In the intervention group, several work accommodations were made from baseline to post-intervention: some individuals changed to a higher function or to more demanding work activities, some to a lower function or less demanding work activities, others did not change.
